# La Grippe: Its Etiology, Clinical History and Treatment*Read before Ga. Medical Association, at Augusta, Ga., April 17, 1891.

**Published:** 1891-10

**Authors:** A. C. Davidson

**Affiliations:** Sharon, Ga.


### Southern Medical Record.

A MONHLY JOURNAL OF MEDICINE AND SURGERY.

VOL. XXI. ATLANTA, GA., OCTOBER, 1891. No. 10

### Original Articles.

### LA GRIPPE: ITS ETIOLOGY, CLINICAL HISTORY AND TREATMENT.*

BY A. C. DAVIDSON, M. D., SHARON, GA.

LaGrippe, so-called, has prevailed almost universally throughout Middle Georgia since about the 20th of December last. Probably 90 per cent. of all the people, white and black, have been attacked.

The majority of cases, however, in my immediate section of country, have been mild, and a small per cent. only have been forced to take their beds. The same disease prevailed to a considerable extent last winter and during the early spring months of last year.

Most of persons attacked last winter have suffered from a second attack this, and in some instances the same individual has suffered from two distinct attacks during the present epidemic. Thus it seems that one attack does not confer immunity from an attack in any future epidemic.

The definition of this disease, as given by Prof. W. J. Conklin, of Dayton, Ohio, is more descriptive of the epidemic as it prevailed last winter than that of this.

He describes it as "a specific, self-limited epidemic fever, characterized by catarrhal inflammation of the mucous mem-

*Read before Ga. Medical Association, at Augusta, Ga., April 17, 1891.

brane of the air passages, and in many cases, also, of the digestive tract; by nervous symptoms and by extreme debility."

Prof. James C. Wilson, of Philadelphia, speaks of it as "a continued fever, occurring in widely extended epidemics, and due to a specific cause; it is characterized by early catarrh of the mucous membrane of the respiratory tract, and in many cases also of the digestive tract; by quickly oncoming debility out of proportion to the intensity of the fever and the catarrhal processes and by nervous symptoms."

He, furthermore, says that "epidemic catarrhal fever" is the most satisfactory of the so-called scientific names by which the disease is at present known.

To assert, and maintain, that the recent epidemic, familiarly known as LaGrippe, has been an epidemic of catarrhal fever, simply, is in my opinion, incorrect. Indeed, by careful attention, I have observed that at least one-half of the persons attacked by this disease this winter have lacked altogether the more prominent symptoms of a catarrh of any kind, if we accept the etymology and derivation of the word as descriptive of the thing described.

Catarrh was more marked, and was far more characteristic of the disease last winter than this.

Last winter nearly every person attacked suffered, during the initial stages, of acute coryza; it has not been the history of the disease this.

While there was a great deal of sneezing, and a vast amount of downward flowing (catarrh) from the nose, together with considerable cough, complicating the disease last year, the two former symptoms have been largely absent this.

Last winter neuralgia of the face and head was a prominent symptom; this winter neuralgia has been less marked, but rheumatism has been more marked. Last year the disease resembled dengue very much; this year it has been more like an abortive attempt at acute inflammatory rheumatism, involving both the muscular and articular system. Last winter the upper air passages, the nasal, post-nasal, laryngeal and tracheal, together with the frontal sinuses and the caves of Highmore, were the more involved, the bronchi, both the larger and the capillary, have been almost universally involved this. Last year gastro-enteric symptoms were rare; this year, especially among the children, gastric and enteric disturbances have been the rule. Icterus, more or less marked, has been observed in most cases this winter; was very rare last. Neurasthenia was a common and persistent sequela last winter; h[illegible]s been r[illegible]re this.

The above are the general differences which have marked the progress and clinical history of LaGrippe last winter and this.

During the prevalence of the epidemic last winter there was a small per cent. of cases which presented symptoms resembling very closely malignant typhoid fever rapidly developed.

In such cases the more prominent symptoms of acute catarrh, such as acute coryza, sneezing, etc., were absent; but there was present a bronchial cough and great muscular soreness, involving nearly all the muscles of the entire body, but mainly the thoracic, the abdominal and lumbar.

There was also present extreme cephalalgia, involving the entire head. These cases were also characterized by obstinate, almost colliquative diarrhœa of characteristic typhoidal color, consistency and fetor.

The tongue presented that peculiar, dry, seared appearance, frequently seen in the more aggravated cases of typhoid conditions.

Abdominal tympanitis, subsultus tendonum, cardiac dicrotism, muttering dilirium, incoherent utterances, coma vigil and great prostration were present during the first few days; and such cases were usually fatal.

There have been a larger per cent. of somewhat similar cases this winter, but of not so malignant a type—indeed, while I have seen quite a score or more of such cases since the outset of the epidemic this winter, I have witnessed only one fatal case.

The two cases seen last winter and the score of cases seen this, were not typhoid fever, as some of my confreres have thought, but were of a malignant type of the same epidemic; sometimes occuring in the same family, and at the same time that other members of the family were sick of the same malady—but of the usual variety—and in sections of the country in which there was no typhoid fever prevailing. Nor can it be said, truthfully, that these cases were of a different disease and greatly modified by the prevailing malady, for the two cases referred to as having occurred last winter, and nearly all the cases so affected this, were apparently in robust health when first attacked by La Grippe, and no history of any prodromata could be obtained by diligent and careful investigation.

It was probably of this type of La Grippe of which the late much lamented Grady died.

I am of the opinion that these cases, presenting such grave typhoidal symptoms, imbibed, ingested or inhaled a greater amount of the peculiar virus which has given rise to this strange disease, protean-like in its many manifestations.

During the present epidemic, there has occurred in my practice a number of cases of this disease which have presented all the characteristic symptoms of pure cholera-morbus. This variety has occurred principally among children, the little patient being suddenly taken with sick stomach and loose bowels, without the usual accompanying soreness and aching of which other unfortunates have complained.

I have noticed that the child thus attacked had very red—sometimes fiery red—lips, abnormally red fauces, and a very clean, but moist, red tongue sparsely covered by small lenticular papillæ. There was also considerable tenderness upon pressure over the stomacic, hepatic and abdominal regions. This variety was marked by great prostration, very frequent pulse, rapid and labored respiration, and subnormal temperature, and in most cases was followed by jaundice more or less marked. So much so that I have been frequently asked if there was not an epidemic of jaundice among the children.

I stated, in the beginning of this paper, that "at least one-half of persons attacked by this disease have lacked altogether the more prominent symptoms of a catarrh of any kind."

This fact has been recalled frequently by the parent announcing: "Doctor, only one, or only two—as the case might be—of my children have a bad cold."

If we admit, as doubtless is true, that catarrhal symptoms have been the leading and most prominent manifestations of

La Grippe—taking the disease last year and this as a whole—we are still at a loss to know what has caused these catarrhal symptoms.

Indeed, we are at a loss to know what has given rise to all this train of various and greatly diversified manifestations noted of this epidemic, as above mentioned.

Catarrh, being derived from the Greek—Katarroos—literally means to flow downward. It is defined as "a discharge of fluid from a mucous membrane, and generally restricted to the mucous membrane of the respiratory tract."

It would naturally follow, therefore, that epidemic catarrhal fever would be characterized, universally, by uniform catarrhal symptoms, at least by the leading or more prominent catarrhal symptoms.

This, we have seen, has not been the case during this present epidemic.

With regard to the etiology of La Grippe, there has been considerable difference of opinion.

In keeping with the views of the more advanced medical bacteriologists of the present day, most medical writers have held, and do hold, that this disease is caused by a specific germ floating in the atmosphere, infinitessimally small and exceedingly numerous—in numbers like unto the far-renowned locusts of old that sometimes infested the ancient countries of the East. Of this theory, however, they have no proof, seeing no one has been able to find and isolate one of these supposed germs. Others, with as much reason, look for its cause in certain meteorological conditions.

If the views of the former be correct, there must have been innumerable quantities of germs permeating the atmosphere of the whole world; for no part of our globe from which we can gain any information has been free from the disease. It is said to have commenced its attack in Russia, but did not long remain peculiar to that inhospitable region, but soon spread itself over the entire habitable world, and for aught we know, over the uninhabitable portions also.

If these supposed germs be things of life, living germs, they are somewhat different from most living things of either the animal or vegetable kingdom, in that they have thrived more luxuriantly in a cold climate than in a warm; and have increased more rapidly in the colder seasons of the year than in the summer time.

Hence we infer that inasmuch as it is entirely contrary to theusual order of nature to propagate and grow living things in a freezing temperature, that this theory of the cause of La-Grippe is incorrect.

Again, we are not sure that any disease is caused by a living germ.

Because pathologists have found bacilli in the sputum, and in diseased lung tissue of tuberculous patients, it is not proof positive that the said baccilli caused the tuberculosis.

Because micrococci are found in great numbers in diphtheretic membranous exudate, it cannot be shown positively that the micrococci caused the diphtheria.

"Wherever the carcass is, there will the eagles be gathered together," is a scriptural trueism, but admirably adapted to modern pathological theory. As a matter of course, there will be no eagles where there is no carcass.

It would be highly unreasonable for us to assert, because we find innumerable quantities of maggots in a piece of putrefying flesh, that the maggots caused the putrefaction. It would be far more reasonable for us to suppose that the maggots were there simply because the flesh was dead and decomposing, and because putrefying flesh is a natural habitat for maggots, both as a nidus for propagation and a pabulum for sustentation and development. The application is easy.

Many modern etiologists seem to have lost sight of every factor in the product of any morbific process except the death-dealing germ.

With them heredity, diatheses, the vicissitudes of weather and other meteorological conditions, elements of decomposition, impure atmosphere, gluttony, insufficient and unwholesome food, inebriety and other environments, potent factors in the formation of disease, are entirely overlooked. Having their gaze fixed upon the one ignis fatuus, they become oblivious to all things else in heaven, earth or sea.

Furthermore, with regard to the etiology of LaGripppe:

I do not think it has been caused by cold alone; but it has been influenced and modified greatly by the various changes in the weather. I have noticed that when the winds have come from a south-easterly, southerly or south-westerly direction, whether the weather was fair, cloudy or rainy, there has been a corresponding amelioration and suspension of the prevalence of the epidemic, both with regard to its progress and type. Fewer persons were attacked and the type was milder.

Twice since the on-coming of the epidemic this winter have I hoped that the malady was becoming exhausted and would soon be a thing of the past, when suddenly, upon a change of the winds to a north-westerly, northerly or north-easterly course, and a corresponding lowering of the temperature, my hopes would be blighted by a revival of the epidemic, both in progress and in severity of type. More especially has this been the case when the winds have blown stiffly from the north-west.

Again, it is possible that LaGrippe may owe its origin to some condition of the atmosphere peculiar to last winter and this; or to some exceedingly attenuated toxical substance of elemental, mineral, earthy or ærolitic nature, floating in space.

Do we know all about the varying conditions of the ether in which we float as we travel through space?

We know that the moon around the earth doth run, and the earth and moon around the sun; but do we know into what portions of the limitless universe of space the sun, with all its coterie of worlds which revolve around it, moves? And do we know what various elemental or other modifying influences this great system encounters, or runs into, and moves through, in its travels along its trackless journey?

In traveling by rail, in the course of a few miles, we may encounter quite a number of varying conditions of atmosphere. The train of cars on which we ride may in one moment run through a shower of rain, the next through a volume of smoke, and again through an atmosphere fragrant with the breath of many flowers.

Are we certain that our system of worlds encounters nothing in the unexplored regions of space in which it moves?

A few years ago—during the winter of 1883 and '84—there was a remarkable phenomenon apparent every afternoon, evening and morning, consisting of a dull, red sky; which at times seemed to cover the entire vault above us There was a great deal of speculation with regard to it, and many opinions were suggested. The wise men of the world said that our system had run into an immense cloud of very fine dust of something similar to the red oxide of iron.

Probably this was true; at any rate it was as good a solution as any other, because nobody knew what caused the phenomenon.

This opinion, with regard to the etiology of LaGrippe, has been suggested, and is supported to a considerable degree by the fact that epidemics, involving nearly the entire world, have made their appearance in irregular cycles returning in periods of time ranging from a few years to nearly a hundred.

The first epidemic of which we have any chronological or descriptive history, occurred in the fourteenth century, and prevailed three successive winters—from 1323 to 1326.

Then there was a skip of 84 years, to 1410; at which time it prevailed, with more or less severity, for four years.

Then comes another skip of 96 years, to 1510. This was the longest period of time intervening between any epidemic. Epidemics of LaGrippe occurred more frequently in the 16th, 17th and 18th centuries than in any others of which we have any record. During those three centuries there were recorded no less than twenty epidemics. These epidemics were not all characterized by the same train of symptoms. Those of the 16th century were spoken and written of as the "epidemic headache."

While we may not be able to say positively what the cause of LaGrippe may be, we can, by carefully observing its many varying symptoms, treat it with certainty of success, and can cure nearly every case of it, notwithstanding it is said to be a self-limited disease.

In the neuralgic form of LaGrippe, accompanied by fever of any consequence, and uncomplicated by either diarrhœa or constipation, I have had abundant success with the following plan: For an adult I order acetanilid, 5 grains every three hours, and until the fever is abated and the neuralgia subdued. Sometimes phenacetin is used in the place of acetanilid. Especially is this latter drug used if the patient is able to purch se it, it being about six times as costly as acetanilid; and when very young children are being treated.

With these two drugs—phenacetin and acetanilid—there is nothing to be desired in treating not only the neuralgic but the rheumatic type also. I have seen case after case—up to many scores of cases—yield in a day or two as if by magic.

Either of these two medicines will not only cure the pain and fever of LaGrippe, but the cough also. This fact has been recognized by quite a number of the laity, who, not knowing the nature of the medicine, speak of it as cough powders.

In the gastro-enteric variety—especially those cases characterized by great nausea and vomiting, and by frequent discharges from the bowels, and by fever, or by a sub-normal temperature—I have commenced treatment by giving an emetic dose of ipecac, fld. ext., and when free from emesis has resulted, follow with

℞ Carbolic acid . . . . . . . . .
Chloroform . . . . . aa 5 drs.
Alcohol . . . . . . . . . . 6 drs.

M. et. Sig.—Ten drops, in an oz. of water every three hours.

Under this plan, I have seen the diarrhæa disappear and the patient improve rapidly.

In cases presenting symptoms of typhoid fever, characterized by all the train of symptoms spoken of in describing this variety of LaGrippe, I have had complete success this winter with the following:

℞ Chlorate of potas. . . . . . . . . . . . . ʒ ij

Dilute. hydro-chlo. acid . . . . . . . f ʒ ij

Water, q. s. ad. . . . . . . . . . . . . . . ℥ viij

M. et. Sig.—One tablespoonful, in an ounce of water, every four hours, to be alternated with

℞ Iodide of potas. . . . . . . . . . ʒ ij

Syr. sarsaparil. com. . . . . . . f ℥ viij

M. et. sig.—One tablespoonful in a third of a glass of water, every four hours.

With these two preparations I generally commence with the chlorate of potas. and hydro-chloric acid solution, first at 6

a. m., the iodide of potassium solution following at 8 a. m. Thus, by alternation the patient gets a dose of medicine every two hours through the day and up to 12 o'clock at night.

As a matter of course if there be high fever—characterized by matutinal remission and vesperal exacerbation, and by very red cheeks, the redness being suffused downward over the face and neck—I have recourse to phenacetin or acetanilid, 4 to 8 gr. doses, every two, three or four hours apart, according to the severity of the fever, and as the case may demand.

I have not found it necessary to give quinine or opium, or any of the derivatives of the latter drug, in combating La-Grippe in any of its varieties.

Indeed, I believe I have done absolute damage to several patients—early in the onset of LaGrippe—last winter by administering to them large doses of quinine. Instead of relieving, it seemed to aggravate the already severe temporal and facial neuralgia.

Again, I believe several deaths have resulted from the injudicious use of morphia in elderly persons who were suffering of LaGrippe in my neighborhood.

It seems to, and doubtless does, only augment the already extreme asthenia which has characterized the larger proportion of cases of this disease occurring in old persons.

One of the cases to which I referred as having occurred last winter, and in whom symptoms of malignant typhoid fever rapidly developed, was a negress, about twenty years old and was apparently in robust health up to the day she was attacked. I was called to see her on the fourth day of her illness. It being one of the first cases which occurred in my practice of this type of the malady, and not being well experienced with the result of morphia in such cases, and in order to control her great restlessness, and also her almost colliquative diarrhœa, I gave her 1-6th grain of morphia per os. The result was, she sank quickly into extreme coma and soon died.

Another which occurred last winter, December 26th, 1889, was that of a robust negro, about thirty years old, to whom I was called on the fourth day of his illness, and in whom neuralgic symptoms were very prominent. I gave him, hypodermically, 1-4th grain of morphia (P. D. & Co.'s tablets.) He sank rapidly into coma and died in about six hours.

From my sad experience with these two cases I learned to be very cautious in theuse of opiates in treating any form of LaGrippe.

